# *Claviplatyshenryi*, a new genus and species of Plataspidae from southern India (Hemiptera, Heteroptera)

**DOI:** 10.3897/zookeys.796.21515

**Published:** 2018-11-15

**Authors:** Dávid édei, Zdeněk indra

**Affiliations:** 1 Institute of Entomology, College of Life Sciences, Nankai University, Weijin Road 94, 300071 Tianjin, China Nankai University Tianjin China; 2 Department of Zoology, Hungarian Natural History Museum, Baross u. 13, H-1088 Budapest, Hungary Hungarian Natural History Museum Budapest Hungary; 3 Department of Plant Protection, Faculty of Agrobiology, Food and Natural Resources, Czech University of Agriculture, CZ-165 21 Praha 6 – Suchdol, Czech Republic Czech University of Agriculture Prague Czech Republic

**Keywords:** Heteroptera, Indomalaya, new genus, new species, Plataspidae, taxonomy

## Abstract

*Claviplatys***gen. n.** and its type species *C.henryi***sp. n.** (Hemiptera: Heteroptera: Plataspidae: Plataspinae) are described from Kerala, India. The new genus is related to the Indomalayan genera *Heterocrates* Amyot & Serville, 1843, *Cratoplatys* Montandon, 1894, and *Cronion* Bergroth, 1891, but differs from them and all other plataspid genera by the peculiarly modified antenna. The morphological characters and systematic relationships of the above genera are discussed.

## Introduction

The family Plataspidae (Hemiptera: Heteroptera) is restricted to the Old World and currently contains in the region 66 genera and 600 species (Rider et al. 2018). With approximately 33 genera and 270 species, Indomalaya accounts for roughly half of the family’s diversity. Faunal study of the region underwent an active period during the late 19^th^ and early 20^th^ centuries; the most prolific authors were A.L. Montandon and W.L. Distant. During the 20^th^ century, only the Chinese fauna was thoroughly explored, due mainly to the activity of W.I. Yang, T.Y. Hsiao and coauthors. The only available comprehensive treatments focusing on larger areas in the region are an outdated revision of the fauna of the former British India, including Ceylon and Burma (Distant 1902, 1908, 1918); two still-usable revisions of the fauna of China (Yang 1934, Hsiao and Jen 1977); and a problematic review of the fauna of Pakistan and neighboring countries, replete with taxonomic confusion (Ahmad & Moizuddin 1992). The fauna of the Indian subcontinent, Indo-China, and Malesia remains in need of a modern revision, with numerous new taxa waiting description.

The purpose of the present paper is to describe a new genus and new species of Plataspidae from southern India. This contribution is dedicated to Thomas J. Henry on the occasion of his 70^th^ birthday, in recognition of his fundamental contributions to a broad variety of heteropteran groups.

## Materials and methods

The specimens on which the present study is based are preserved in the Hungarian Natural History Museum (**HNHM**) and in Zdeněk Jindra’s personal collection, currently deposited at the Department of Plant Protection, Czech University of Agriculture, Prague, Czech Republic (**ZJPC**). External structures and genitalia were examined using a stereoscopic microscope (Zeiss Discovery V8). Drawings were made with the aid of a camera lucida. Genitalia of both sexes were dissected after careful heating in hypertonic KOH solution, cleared with lactophenol, and slightly stained with Chlorazole Black E. Measurements were taken using a micrometer eyepiece. Digital photographs were taken with a Nikon D90 camera equipped with AF-S Micro Nikkor 60mm f/2.8G ED macro lens. Morphological terminology mainly follows Tsai et al. (2011), Rédei and Jindra (2015), and Rédei (2016, 2017a).

## Taxonomy

### Claviplatys gen. n.

Taxon classificationAnimaliaHemipteraPlataspidae

http://zoobank.org/8DCC7784-8622-4328-AD90-5C6F5A9F1091

Figures 1–4 , 5–9

#### Type species by present designation.

*Claviplatyshenryi* sp. n.

#### Diagnosis.

**Figures 1–4.**
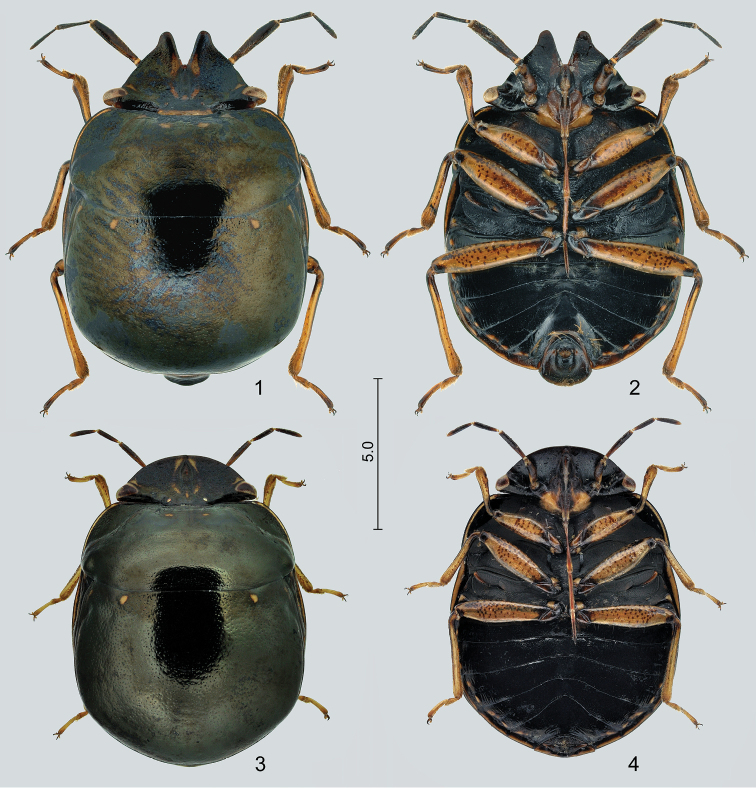
*Claviplatyshenryi* gen. et sp. n. **1** holotype (male), dorsal view **2** same, ventral view **3** paratype (female), dorsal view **4** same, ventral view. Scale bar in mm.

Medium-sized plataspids with weakly convex dorsum and flat venter (Figs 1–4); head strongly broadened and flattened, width ca. 70% of width of pronotum, strongly sexually dimorphic, mandibular plates of male produced much anteriad of apex of anteclypeus, not adjacent, separated by V-shaped interspace, those of female produced slightly anteriad of apex of clypeus, narrowly overlapping, forming evenly rounded outline anteriorly; interocellar distance distinctly shorter than (♂) or subequal to (♀) distance between ocellus and ipsilateral eye; scape thick, distipedicellite strikingly (♂) or considerably (♀) broadened and flattened, anterior surface with long, erect hairs (some longer than diameter of segment), basi- and distiflagellum much thinner than distipedicellite (Figs 6–9). The unique, peculiarly modified antenna is diagnostic for the genus.

#### Description.

***Body*** (Figs 1–4) medium sized (approx. 10–12 mm), broadly oval, dorsum relatively weakly convex, venter flat; dull black with ochraceous markings. ***Integument and vestiture.*** Body at most with fine, dense, superficial, inconspicuous punctation, occasionally finely rugose on some body parts; virtually glabrous dorsally and ventrally, appendages with short, inconspicuous, adpressed or semierect pilosity, distiflagellum with several conspicuously long and erect setae on anterior surface (Figs 6–9).

**Figures 5–15.**
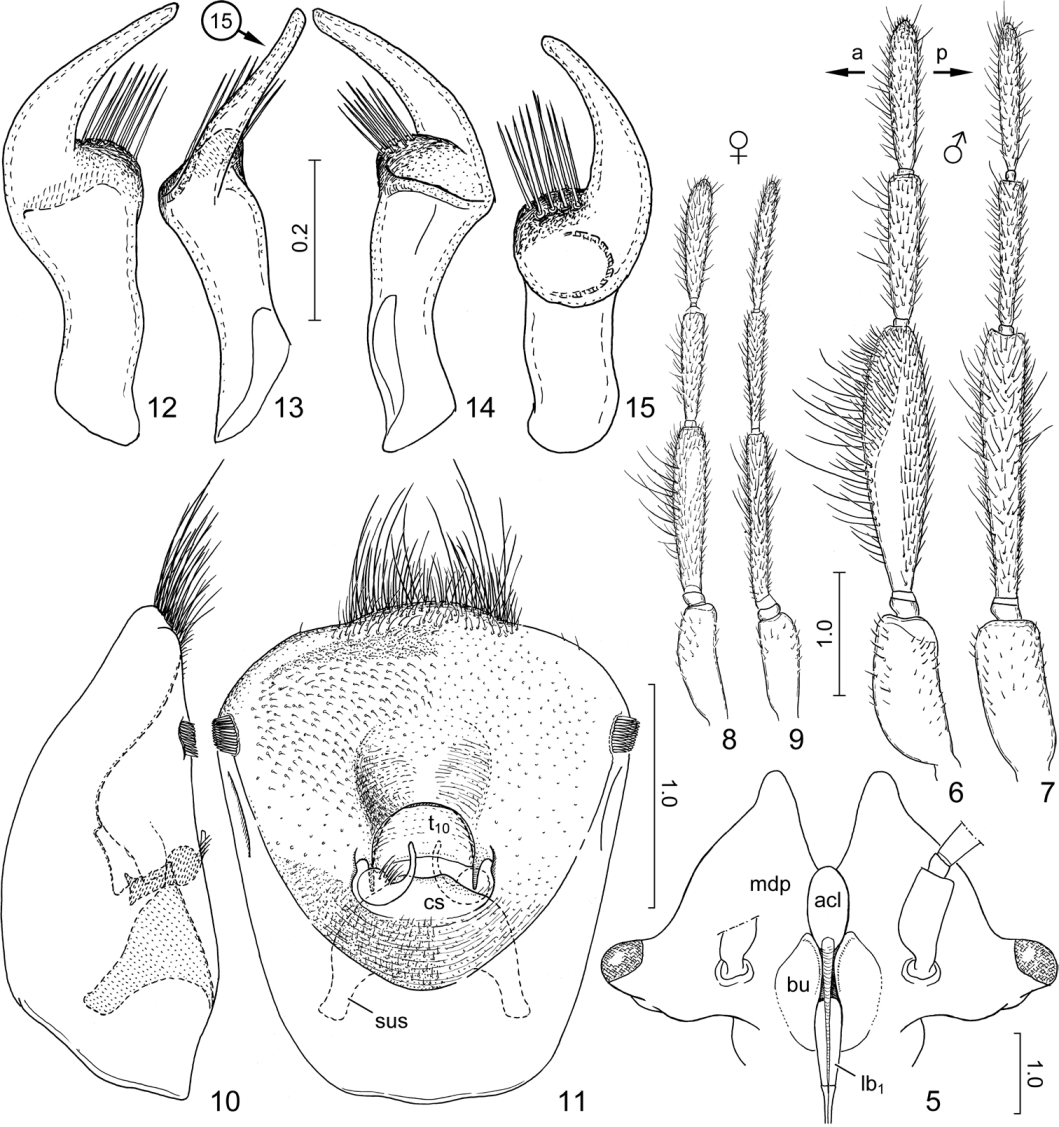
*Claviplatyshenryi* gen. et sp. n., holotype. **5** head, ventral view **6** left antenna of male, ventral surface **7** same, anterior surface **8** left antenna of female, ventral surface **9** same, anterior surface **10** genital capsule, left lateral view **11** same, morphological posterior surface in most exposed view **12–15** left paramere, four different aspects. Arrows in Fig. 6 indicate anterior (a) and posterior (p) direction, that in Fig. 13 shows aspect of Fig. 15. Abbreviations: acl = anteclypeus; bu = buccula; cs = cuplike sclerite; lb_1_ = labiomere I; mdp = mandibular plate; sus = suspensory apodeme; t_10_ = tergite X. Scale bars in mm.

***Structure.****Head and cephalic appendages. Head* much broader than long, 0.7 times as broad as width of pronotum; eyes strongly transverse, strikingly protruding from outline of head laterally (♂) or more rounded, less protruding (♀) in dorsal view; anteclypeus dorsally rather flat, very slightly elevated above plane of mandibular plates; mandibular plates of male (Figure 5: mdp) flattened, strongly produced anteriad far surpassing apex of anteclypeus but not adjacent anteriad of it, portion anteriad of apex of anteclypeus strongly curved upwards (♂), those of female produced slightly anteriad of apex of clypeus, narrowly overlapping, forming evenly rounded outline anteriorly; ocelli close to midline, interocellar distance distinctly longer than (♂) or subequal to (♀) distance between lateral margin of ocellus and mesal margin of ipsilateral eye; antennae inserted on strongly protruding ring-like tubercle slightly closer to base of labium than to mesal margin of eye; bucculae (Figure 5: bu) broad, dorsoventrally flattened. *Antenna* (Figs 6–9): scape thick, cylindrical, subequal in length to the two segments of flagellum; basipedicellite very short; distipedicellite strikingly (♂) or distinctly (♀) broadened and dorsoventrally flattened, basi- and distiflagellum much thinner than distipedicellite, subequal in length, dorsoventrally flattened. *Labium* inserted slightly posteriad of middle of buccula, posteriad of level of posterior margin of eyes; without peculiar modifications, labiomere I (Figure 5: lb_1_) thicker than remaining segments but not conspicuously thickened, labiomere II distinctly flattened laterally, remaining labiomeres subcylindrical.

***Thorax and thoracic appendages.****Pronotum* moderately declivous anteriorly; anterior collar narrow, weakly demarcated; lateral margin broadly, laminately explanate, anterior margin of explanated portion almost straight, slightly produced anteriorly at anterolateral angles, lateral margin evenly convex, gradually broadened posteriad; humeri with small, inconspicuous tubercle; posterior margin broadly rounded, posterolateral angle obsolete. *Scutellum* rather evenly rounded without conspicuous angulations, basal tumescence not elevated and not demarcated by furrow, dorsal outline of scutellum continuous in lateral view; area around basolateral angle not delimited by furrow; with fine submarginal impression along almost entire length except extreme base; posterior margin slightly emarginate above genital capsule. *Thoracic pleura and sterna.* Proepisternum broadly elevated anteriad of proacetabula, prothorax deeply depressed along meson; mesosternum elevated, forming broad, obtuse carina, posterior margin V-shaped and produced between mesacetabula; metapleurite with well-developed, elongate scent gland ostiole closer to dorsal margin of metapleurite than to base of mesocoxa, associated with elongate, slightly anteriorly curved peritreme; metasternum flat, metacoxae close to each other; evaporatorium occupying almost entire ventral surface of thorax except extreme lateral margin of prothorax and small subtriangular area at posterodorsal angle of metapleuron. *Fore wing.* Exocorium and adjacent elongately triangular basal portion of mesocorium exposed at rest. *Legs* short, femora thick, tibiae with distinct, broad, deep dorsal furrow along almost entire length, terminating subapically.

***Pregenital abdomen*** much broader than long; dorsal laterotergites fused into single, undivided synlaterotergite; ventral laterotergites and sternites (= mediosternites) distinctly separated, intersegmental sutures extending to lateral margin of abdomen; ventral laterotergites separated from sternites by deep longitudinal furrow, highly obliquely elevated anteriorly, gradually becoming lower posteriorly, in segment VII coplanar with sternite; spiracles at lateral margin of ventrites; trichobothria in longitudinal furrow between ventral laterotergites and meditergites, posteriad of spiracle of same segment, arranged longitudinally; anterior margin of sternite VII deeply, subtriangularly produced anteriad, reaching anterior margin of ventrite VI medially, thus completely bisecting ventrite VI into two hemiventrites (♂) or only slightly invading ventrite VI posteriorly, with mesal length of ventrite VI approx. two thirds of that of ventrite V (♀).

***External male genitalia.*** Genital capsule relatively small (width approx. one third of width of head), posterior aperture directed ventrad.

***External female genitalia.*** Exposed portions of ovipositor directed ventrad (Figure 21).

#### Etymology.

The generic name alludes to the peculiarly modified antenna of the male, which is diagnostic for this new genus; it is composed of the Latin noun *clava* meaning a club, cudgel, knotty branch or stick and the Greek adjective *πλατύς* (Latinized as *platys*) meaning wide, broad, a component occurring in several generic names in the family Plataspidae. Gender masculine; stem *Claviplate*-.

#### Diversity and distribution.

The single included species occurs in the Malabar region of southern India.

### Claviplatys henryi sp. n.

Taxon classificationAnimaliaHemipteraPlataspidae

http://zoobank.org/01387685-0D74-469F-BA86-71B07E377C5B

Figs 1–4 , 5–15 , 16–20 , 21–23

#### Type material.

**Holotype** (Figs 1–2): ♂, India: Kerala, Pompa [= Pamba], Sabramila [= Sabarimala], 09°24.9'N, 77°03.9'E, 3.v.2005, leg. M. Halada; mounted on card, intact, genitalia detached, preserved in plastic microvial with glycerol; deposited in ZJPC. **Paratypes**: South India: Kerala, Cardamom Hills, 50 km NW of Pathanamthitta, near Pambaiyar River, 9°25'N, 77°05'E, 6–9.v.1994, leg. Z. Kejval (1 ♀ ZJPC, 1 ♀ HNHM).

#### Diagnosis.

*Claviplatyshenryi* sp. n., the single known species of the genus, can be recognized by the diagnostic characters provided for the genus.

#### Description.

**Male** (Figs 1–2, 5–7, 10–20).

***Color.*** Dorsum and venter dull black, with ochraceous markings as follows: pair of longitudinal vittae submarginally along mesal margin of mandibular plates (indistinct around middle), and small spot between proximal extremities of above-mentioned vittae; pair of short, obliquely transverse streaks before mesal angles of eyes; proximomesal portion of ventral surface of clypeus; bucculae and surrounding areas between antennal insertions and basal neck-like portion of head ventrally; pair of small submedian spots on callar lobe of pronotum; pair of narrow submarginal vittae along lateral margin of pronotum, terminating slightly anteriad of humeral tubercle; undulating vitta connecting lateral extremities of anterior collar with humeral tubercles, following mesal margin of lateral explanate lobe of pronotum (indistinct around middle); pair of small sublateral spots on scutellum very close to anterior margin; narrow submarginal vitta around scutellum except extreme base; patch on metapleuron immediately anteriad of scent gland ostiole; distal (= lateral) portion of peritreme; small, rounded spot at posterolateral angle of metapleurite; dorsal laterotergites and adjacent lateral margins of ventral laterotergites of abdominal segments III–VII; pair of longitudinally elongate spots on each mediosternite of segments III–VII marginally (surrounding respective spiracles), and pair of smaller spots posteriad of the above ones on segments III–VI. Scape ochraceous, irregularly suffused with brown especially in distal half, pedicel, basi- and distiflagellum blackish brown; labrum ochraceous. Legs ochraceous, coxae with large dark brown patch on mesal surfaces; trochanters broadly suffused with dark brown distally, femora with several rounded brown patches on (morphological) anterior and posterior surfaces, with blackish brown longitudinal streak dorsoapically and black patches on anterior and posterior surfaces ventroapically, tibiae with few small, rounded, brown patches and longitudinal streak on nearly entire length of dorsal (furrowed) surface, tarsal segments suffused with brown apically.

***Integument and vestiture*** generally as in generic description. Sternite VI with pair of fringes of long setae arranged along curved line following posterior margin of segment; dorsum of mandibular plates irregularly rugose, pronotum and scutellum with fine, dense, superficial, inconspicuous punctation, abdominal ventrites finely longitudinally wrinkled.

***Structure.****Head* 1.85 times as broad as medial length (from base to imaginary line connecting tips of mandibular plates), 1.5 times as broad as interocular distance; mandibular plates (Fig. 5: mdp) produced anteriad as pair of subtriangular, apically narrowly rounded plates, mesal margins almost straight; distance between ocellus and ipsilateral eye 1.2 times as long as interocellar distance. *Antenna* (Figs 6–7): distipedicellite approx. four times longer than greatest width, 1.75 and 1.65 times longer than basipedicellite and distipedicellite, respectively. *Labium* reaching base of abdominal sternite V.

***Thorax*** and ***pregenital abdomen*** as described for genus. Prothorax 2.2 times, scutellum 1.5 times broader than median lengths of respective sclerites.

**Figures 16–20.**
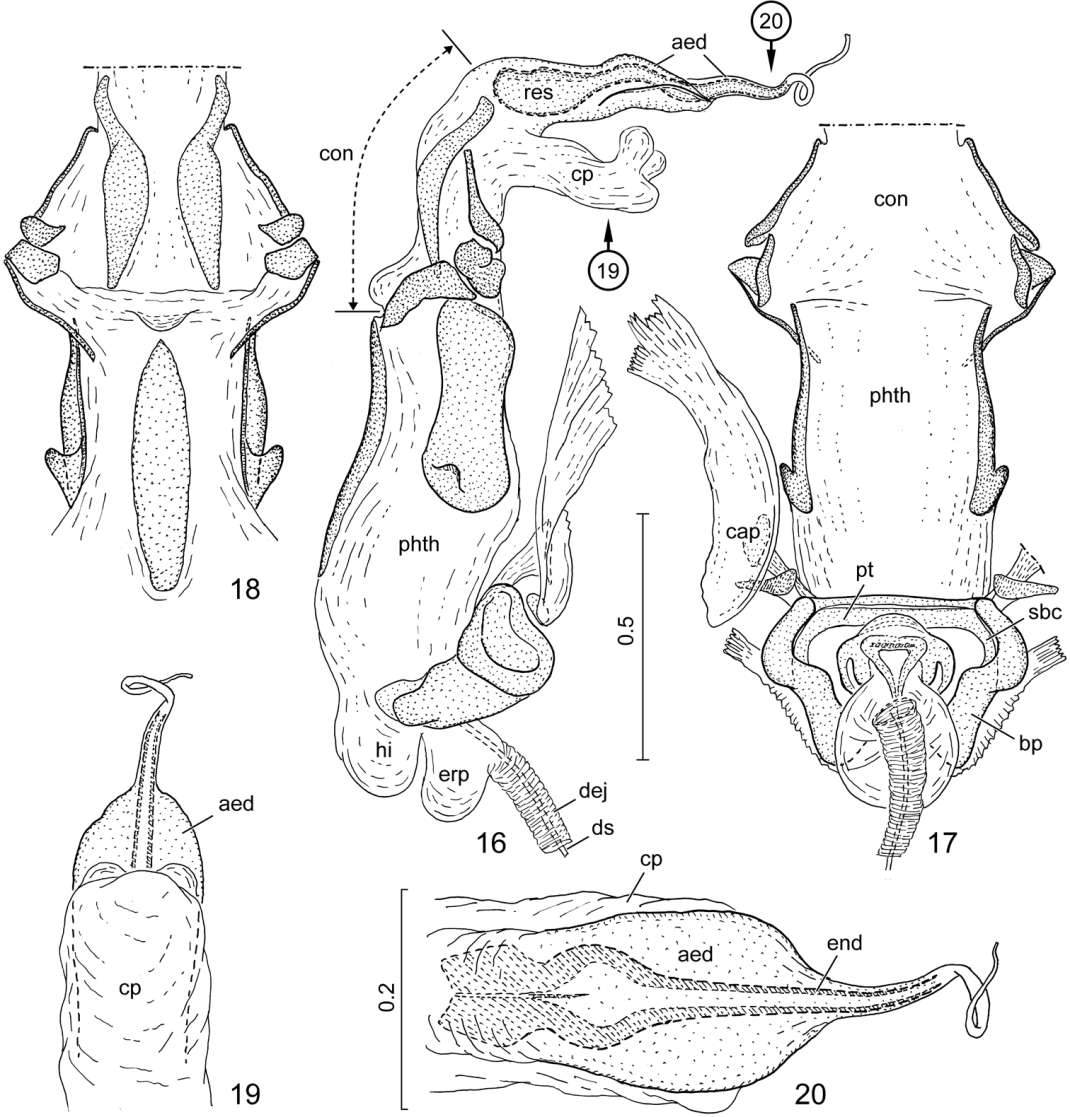
*Claviplatyshenryi* gen. et sp. n., holotype. **16** phallus, lateral view **17** same, dorsal view (conjunctival process omitted, aedeagus not shown) **18** same, ventral view (articulatory apparatus and aedeagus not shown) **19** conjunctival process and aedeagus, morphological dorsal view **20** same, morphological ventral view. Arrows in Fig. 16 show aspect of Figs 19 and 20. Abbreviations: aed = aedeagus; bp = basal plates; cap = capitate process; con = conjunctiva; cp = conjunctival process; dej = ductus ejaculatorius; ds = ductus seminis; end = endophallic duct; erp = erection fluid pump; hi = hinge; phth = phallotheca; pt = ponticulus transversalis; res = endophallic reservoir; sbc = support bridge complex. Scale bars in mm.

***External male genitalia.****Genital capsule* (Figs 10–11) broadly oval, posterior margin broadly rounded, slightly protruded posteriorly in mesal third, protruded part with tuft of several long, erect setae; lateral margin with pair of small, compact, brush-like clusters of strong, stiff setae subapically; morphological posterior surface weakly concave, infolding of dorsal rim broad, broadly and deeply impressed medially immediately dorsad of posterior aperture; infolding of ventral rim relatively broad; posterior aperture small, dorsal sinus broadly rounded, completely occupied by tergite X (Figure 11: t_10_); cuplike sclerite (Figure 11: cs) completely fused with infolding of ventral rim of genital capsule without trace of fusion line, provided with pair of thick suspensory apodemes (Figure 11: sus). *Paramere* (Figs 12–15) sickle-shaped, with short crown comprising broad stem and elongate, narrow apical process. *Phallus* (Figs 16–20): basal plates (Figure 17: bp), robust, fused with support bridge complex (Figure 17: sbc) by leaving pair of window-like interspaces open at two sides of median portion of support bridge complex, support bridge complex with broad ponticulus transversalis (Figure 17: pt); phallotheca (Figs 16–17: phth) elongate, greatly membranous, with protruding, diverticulum-like hinge (Figure 16: hi) proximoventrally, with pair of dorsolateral and single ventromedian sclerite distally, each dorsolateral sclerite with blunt, rounded, posteriorly directed tubercle at proximal part; conjunctiva elongate, wall of proximal portion with 4 pairs of sclerites: pair of elongate sclerites submedially, and three pairs of sclerites (derivatives of conjunctival processes?) laterally, apparently forming complex articulation for supporting proximal part of conjunctiva; distal portion with single elongate, membranous, tubular process dorsally (Figs 16, 19, 20: cp) provided with pair of very short, rounded, membranous diverticula subapically on posterior surface; aedeagus (Figs 16, 19, 20: aed) indistinctly demarcated from conjunctiva, weakly sclerotized, somewhat dorsoventrally compressed, produced into pair of flattened, fin-like expansions laterally, abruptly narrowed around middle, gradually tapering towards apex; endophallic reservoir elongate, endophallic duct thin, tubular, rather weakly sclerotized, gradually tapering to distal third, then desclerotized and closely surrounded by fine, transparent outer wall of aedeagus, forming thin, flexible tube.

**Figures 21–23.**
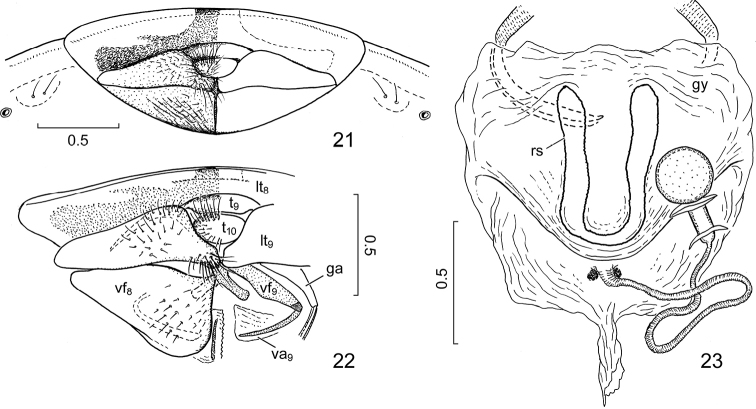
*Claviplatyshenryi* gen. et sp. n., external female genitalia. **21** terminalia, ventral view **22** same, dissected, right valvifer VIII removed (drawn after macerated preparatum) **23** gynatrium and spermatheca, dorsal view after removal of abdominal dorsum. Abbreviations: ga = gonangulum; gy = gynatrium; lt_8_, lt_9_ = laterotergites VIII and IX; rs = ring sclerite; t_9_, t_10_ = tergites IX and X; va_9_ = valvula IX; vf_8_, vf_9_ = valvifers VIII and IX. Scale bars in mm.

**Female** (Figs 2–3, 21–23). ***Color, integument, and vestiture*** as in male. Terminalia brown, with long hairs on laterotergite IX and tergite X forming conspicuous, brush-like tuft around midline.

***Structure.****Head* 2.3 times as broad as medial length, 1.5 times as broad as interocular distance; anterior outline evenly rounded, mandibular plates narrowly overlapping anteriad of apex of clypeus; distance between ocellus and ipsilateral eye subequal to interocellar distance. *Antenna* (Figs 8–9): distipedicellite 5.5 times longer than greatest width, 1.35–1.5 and 1.2–1.25 times longer than basipedicellite and distipedicellite, respectively. *Labium* reaching base of abdominal sternite V.

***Thorax*** and ***pregenital abdomen*** as in male. Prothorax 2.2 times, scutellum 1.4 times broader than median lengths of respective sclerites.

***External female genitalia*** (Figs 21–23). Laterotergites VIII (Figure 22: lt_8_) not separated along midline, forming broad transverse plate posteriad of laterotergites IX; valvifers VIII (Figure 22: vf_8_) relatively short, subequal in length along midline as laterotergites IX (Figure 22: lt_9_); valvifers IX (Figure 22: vf_9_) obliquely elongate, with pair of shorter, finger-like, oblique processes mesally; gynatrium (Figure 23: gy) simple, saccular, lacking distinct pouches, with broad fold immediately proximad of orifice of spermatheca, contralateral ring sclerites fused along midline into single clearly sclerotized and easily traceable U-shaped sclerite (Figure 23: rs); with pair of small, rounded sclerites at two sides of spermathecal orifice; spermathecal duct simple, thin, intermediate part of spermatheca relatively short, apical receptacle globose.

#### Measurements

**(in mm).** Male (n = 1) / female (n = 2): Body length measured along meson from imaginary line connecting tips of mandibular plates to apex of scutellum (♂) 11.6, to apex of genital capsule in dorsal view (♂) 12.2, from apex of head to apex of scutellum (♀) 10.0–10.1; length of head measured along meson from base to imaginary line connecting tips of mandibular plates (♂) 3.00, from base to anterior margin (♀) 2.06–2.08, greatest width across eyes 5.55 / 4.75–4.80, interocular distance 3.75 / 3.20–3.23, interocellar distance 1.05 / 1.00–1.02, distance between mesal margin of eye and ipsilateral ocellus 1.25 / 1.00–1.00; length of scape 1.13 / 0.93–0.95, greatest width 0.50 / 0.26–0.28, length of basipedicellite 0.18 / 0.09–0.10, greatest width 0.27 / 0.15–0.16, length of distipedicellite 2.00 / 1.20–1.20, greatest width 0.50 / 0.20–0.22, length of basiflagellum 1.13 / 0.80–0.90, greatest width 0.21 / 0.15–0.16, length of distiflagellum 1.20 / 0.96–1.00, greatest width 0.20 / 0.16–0.17; length of labiomere I 1.10 / 0.98–1.00, II 2.00 / 1.74–1.85, III 1.48 / 1.32–1.40, IV 1.13 / 1.10–1.15; length of pronotum along meson 3.62 / 3.15–3.17, greatest width 7.94 / 7.06–7.08, length of scutellum along meson 5.73 / 5.36–5.54, greatest width 8.51 / 7.50–7.56.

#### Etymology.

Patronymic, dedicated to Thomas J. Henry.

#### Distribution.

Known only from the Malabar Subregion of southern India. The holotype and the two paratypes were collected at two localities around Pamba station below Sabarimala, a Hindu pilgrimage center, in Pathanamthitta District, Kerala State, separated by a distance of ca. 2 km.

## Discussion

*Claviplatys* gen. n. is placed in Plataspinae, more precisely in the narrowly defined Plataspinae corresponding with the *Brachyplatys* group of authors (Jessop 1983, Rider et al. 2018). It is a member of a well-recognizable, presumably monophyletic group of genera (herein, “*Heterocrates* group”), including *Heterocrates* Amyot & Serville, 1843 (Malay Archipelago), *Cratoplatys* Montandon, 1894 (Indo-China), and *Cronion* Bergroth, 1891 (Malay Peninsula). *Codronchus* Distant, 1901, from the Andaman Islands, not examined during the present study, potentially belongs to this group. The *Heterocrates* group can be defined by the combination of the following characters (supposed synapomorphies marked by an asterisk):

(1) ground color black, usually decorated by ochraceous spots and strikes at least along lateral margins of prothorax and abdomen and costal margin of fore wing,

(2) body strongly flattened, dorsum weakly convex, venter flat,

(3*) head sexually dimorphic,

(4*) head greatly broadened, width at least approx. 70% of that of prothorax,

(5*) postocular margin of head nearly straight between level of ocellus and eye,

(6*) mandibular plates strongly flattened, laminate, surpassing tip of anteclypeus anteriorly,

(7*) eyes small, placed strongly laterally,

(8*) ocelli close to midline and far removed from mesal margins of compound eyes (distance between ocellus and ipsilateral eye distinctly greater than interocellar distance),

(9*) antennal insertion far from compound eyes,

(10*) anterior margin of pronotum weakly emarginate,

(11*) basal tumescence of scutellum not elevated and not demarcated by furrow, dorsal outline of scutellum continuous in lateral view,

(12*) posterior aperture of genital capsule (♂) and ovipositor (♀) directed ventrally.

The *Heterocrates* group apparently is related to *Brachyplatys* Boisduval, 1835 (Afrotropical, Indomalayan and Australian Regions). The latter genus is more generalized than are members of this group, and it shares most of the characters listed above, except characters 3 (head not sexually dimorphic) and 8 (ocelli much closer to eyes than to each other). Furthermore, in the majority of *Brachyplatys* species the head is narrower (approx. 60% of width of prothorax), although in some species this value is > 70%; a few species of *Brachyplatys* (e.g. *B.raffrayi* Montandon, 1897 and *B.macrosignatus* Yang, 1932) also are differently colored. *Neocratoplatys* Miller, 1955 (Indo-China) is also superficially similar to members of the *Heterocrates* group, but it is also more generalized because the basal tumescence of the scutellum is delimited by a rather distinct furrow.

Within the *Heterocrates* group, the genera most similar and probably phylogenetically most closely related to *Claviplatys* gen. n. are *Cronion* and *Cratoplatys*; these genera exhibit a particularly striking sexual dimorphism in shape of the head. *Cronion* (recently treated by Rédei 2017b) and *Cratoplatys* share the posteriorly displaced labial insertion and the dorsally flat anteclypeus (both are apparently plesiomorphies in the clade as they also are found in *Brachyplatys* and *Heterocrates*), but in *Cronion* the antenna is relatively slender, the scape and the distipedicellite are subequal in length, the distipedicellite is only slightly thicker than the flagellomeres, and the distipedicellite and flagellomeres are only feebly flattened in both sexes. *Cratoplatys* differs from both genera in having the labium inserted at the anterior extremity of the buccula and the dorsally convex anteclypeus elevated above the plane of the mandibular plates; the antenna in the latter genus is similar to the condition found in *Cronion*, but the scape is distinctly longer than the distipedicellite. The head of both *Cronion* and *Cratoplatys* is broader than in *Claviplatys* gen. n.; in males the width of the head is approx. 80% of the width of the pronotum.

## Supplementary Material

XML Treatment for Claviplatys

XML Treatment for Claviplatys henryi
